# Rapid therapeutic response in refractory lichen amyloidosis treated with upadacitinib: a case report

**DOI:** 10.3389/fmed.2026.1932492

**Published:** 2026-09-22

**Authors:** Yan Teng, Jing Luo, Jiamin Wen, Qiong Gao, Yang Ding, Gairong Liu

**Affiliations:** 1 Department of Dermatology, Tongxiang First People's Hospital, Tongxiang, Zhejiang, China; 2 Center for Plastic and Reconstructive Surgery, Department of Dermatology, Zhejiang Provincial People's Hospital, Affiliated People's Hospital, Hangzhou Medical College, Hangzhou, Zhejiang, China; 3 Department of Dermatology, People's Hospital of Tiantai County, Taizhou, China

**Keywords:** JAK1 inhibitor, lichen amyloidosis, primary localized cutaneous amyloidosis, pruritus, upadacitinib

## Abstract

Lichen amyloidosis (LA) is an intractable, chronic pruritic skin disorder characterized by amyloid deposition within the dermal papillae. It manifests as severely itchy, hyperpigmented papules and cobblestone plaques mainly distributed on the extremities. Conventional treatment modalities often yield partial or temporary therapeutic effects with unstable efficacy. This paper describes the case of a 60-year-old Chinese male patient presenting with a 3-year history of progressive, refractory lichen amyloidosis involving bilateral limbs. Over a 2-year period, the patient had failed multiple successive and combined treatment regimens, including oral antihistamines and potent topical glucocorticoids, while histopathological staining results confirmed primary localized cutaneous amyloidosis. After monotherapy with 15 mg oral upadacitinib once daily, the patient achieved rapid remission: pruritus was greatly relieved within 1 month, with the numerical rating score dropping from 8 to 1–2; this improvement was accompanied by obvious flattening of the papules, a decrease in lesion quantity, fading of pigmentation, and the disappearance of cobblestone skin lesions. No short-term adverse reactions were observed during medication. This case adds new clinical evidence supporting the therapeutic value of JAK1 inhibitors in the management of refractory inflammatory dermatoses, confirms the key involvement of IL-31-dependent JAK1 signaling in the pathogenesis of LA, and highlights upadacitinib as a promising rescue regimen for conventional treatment-resistant lichen amyloidosis, offering clinicians a clinical reference for employing JAK1-targeted therapy to disrupt the pathological itch–scratch cycle. Nonetheless, larger prospective cohort studies and randomized controlled trials are still required to validate the long-term efficacy, optimal dosage, and safety profile of upadacitinib in patients with lichen amyloidosis.

## Introduction

Primary localized cutaneous amyloidosis (PLCA) is a chronic non-systemic skin disorder characterized by amyloid deposition in the papillary dermis. Lichen amyloidosis (LA), the most common subtype, presents as severely pruritic hyperpigmented papules and cobblestone-like plaques predominantly on the extremities (1). Clinically, lichen amyloidosis needs to be differentiated from a spectrum of pruritic hyperpigmented dermatoses, including hypertrophic eczema, hypertrophic lichen planus, nodular prurigo, lichen simplex chronicus, and macular amyloidosis. Histopathological examination is crucial for definitive diagnosis, as it demonstrates dermal amyloid deposits and helps rule out clinically mimicking conditions. Intractable pruritus drives a self-sustaining itch–scratch cycle, and conventional therapies often yield incomplete or transient responses. Treatment remains challenging due to refractoriness and inconsistent efficacy of existing agents (2). Upadacitinib, a selective oral JAK1 inhibitor, has shown promise in many inflammatory skin diseases. We report a 60-year-old man with a 3-year refractory LA resistant to antihistamines and high-potency topical corticosteroids. After starting upadacitinib at a dose of 15 mg daily, pruritus and cutaneous lesions improved rapidly and markedly within 1 month, with excellent tolerability. This case demonstrates that upadacitinib is an effective and safe salvage therapy for recalcitrant LA and supports JAK1-mediated signaling as a key target in its pathophysiology.

## Case presentation

A 60-year-old Chinese male presented to the dermatology clinic with a 3-year history of progressively worsening pruritic papules and plaques on bilateral upper and lower extremities. He described the pruritus as constant, severe, and predominantly nocturnal, with marked disruption of sleep, daily activities, and overall quality of life. He denied any systemic symptoms including weight loss, fatigue, dyspnea, abdominal pain, or peripheral neuropathy. He also denied oropharyngeal or other mucosal lesions. There was no family history of amyloidosis, atopy, or other autoimmune skin disorders. Physical examination revealed symmetrically distributed, monomorphic 1–3 mm brown to hyperpigmented lichenoid papules across the forearms, lower legs, ankles, and thighs. Many papules had coalesced into ill-defined, cobblestone-like plaques with variable scaling, excoriations, and secondary post-inflammatory hyperpigmentation (Figure 1a). Lesions affected approximately 25% of the total body surface area, with relative sparing of the trunk. Pruritus severity was scored 8 out of 10 on a numerical rating scale (NRS 0–10). The patient had received multiple sequential and combination therapies for more than 2 years prior to presentation, all with unsatisfactory results: oral antihistamines (continuous combination therapy with cetirizine 10 mg daily plus loratadine 10 mg daily for 12 months, with minimal reduction in pruritus), topical treatments (high-potency topical corticosteroids including halometasone cream and clobetasol propionate ointment applied twice daily for 8 consecutive weeks, leading to mild temporary relief but no objective improvement in lesion texture, color, or thickness), and adjuvant measures (short courses of cold compresses and emollients, which provided negligible symptomatic benefit). No improvement in pruritus or skin lesions was sustained despite adherence to the aforementioned treatments. To confirm the diagnosis and exclude systemic disorders, comprehensive laboratory and histopathological assessments were performed. Routine laboratory tests, including complete blood count, liver and renal function tests, thyroid function panel, fasting blood glucose, serum immunoglobulin E, serum tryptase, serum protein electrophoresis, and urinalysis, were all within normal reference ranges. A 4-mm punch biopsy from a representative lesion on the right lower leg revealed hyperkeratosis, epidermal acanthosis, and amorphous eosinophilic deposits filling the dermal papillae (Figure 2). The deposits were strongly positive for Congo red and periodic acid–Schiff with diastase (PAS-D) staining, consistent with amyloid deposition. No evidence of systemic amyloidosis or other inflammatory dermatoses was identified. Systemic screening, including chest radiography and abdominal ultrasonography, was unremarkable, effectively excluding visceral involvement. A definitive diagnosis of lichen amyloidosis (primary localized cutaneous amyloidosis) was established. After thorough discussion with the patient, obtaining written informed consent, and excluding contraindications (active infection, severe hepatic dysfunction, uncontrolled hypertension, or history of malignancy), oral upadacitinib at a dose of 15 mg once daily was initiated as monotherapy. No other systemic or topical immunomodulatory agents were administered concurrently except basic emollients. At 2 weeks, subjective pruritus began to improve, with the NRS score decreasing from 8 to 4; no adverse events were reported. At 1 month, pruritus was markedly alleviated (NRS 1–2/10), sleep quality returned to normal, and objective clinical improvement was prominent (cutaneous papules significant flattening, lesion density substantially reduced, hyperpigmentation noticeably lightened, cobblestone-like texture largely resolved, no new lesions developed) (Figure 1b). Safety profile: the patient tolerated upadacitinib exceptionally well throughout treatment, with no reports of headache, nausea, elevated liver enzymes, infection, hematologic abnormalities, or other adverse reactions, while laboratory monitoring at 1 month remained unremarkable. At the time of reporting, the patient continues maintenance therapy with upadacitinib at 15 mg daily and maintains sustained clinical improvement without relapse.

**Figure 1:**
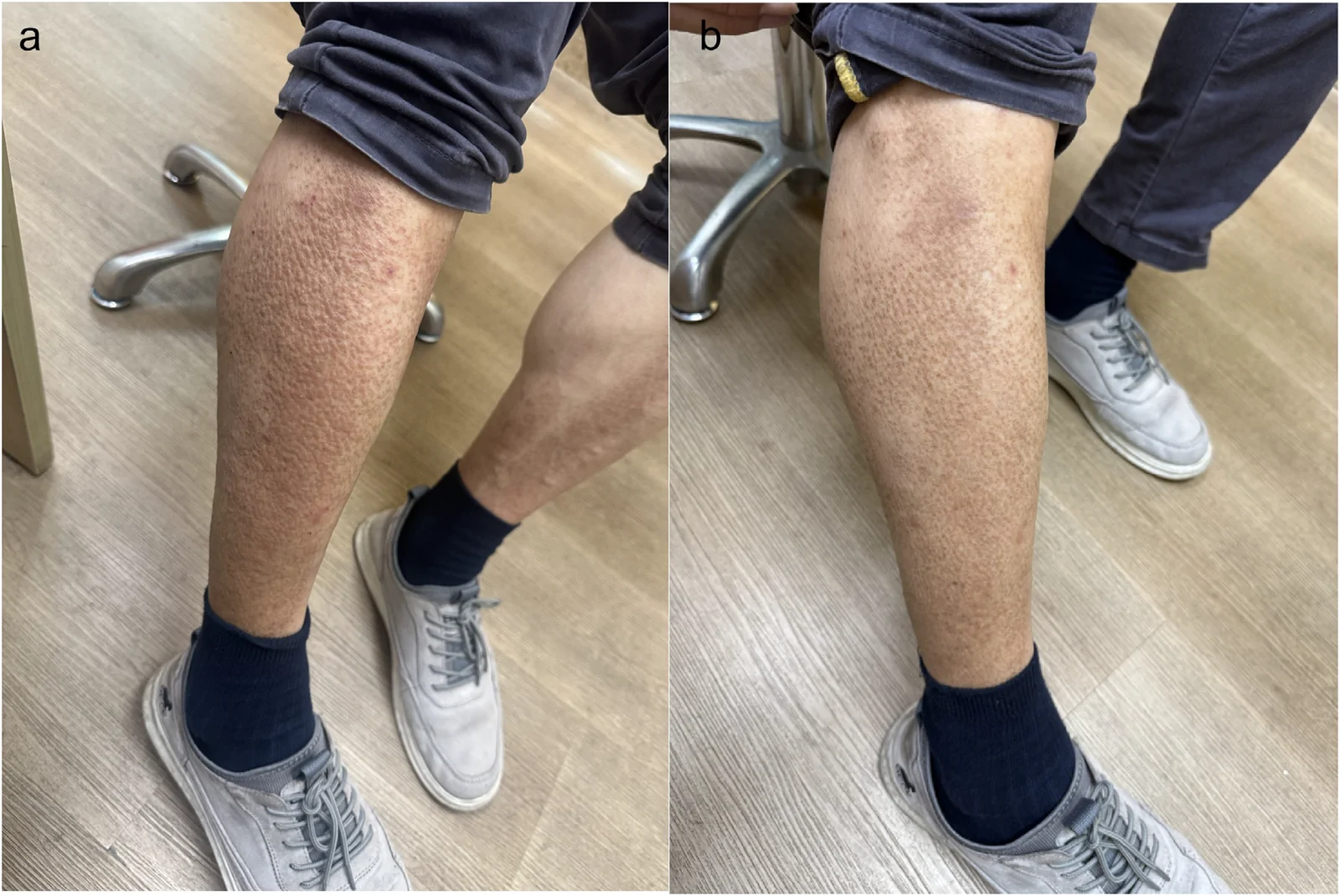
Image of the patient **(a)** at the baseline **(b)** after 1 month of treatment with upadacitinib.

**Figure 2:**
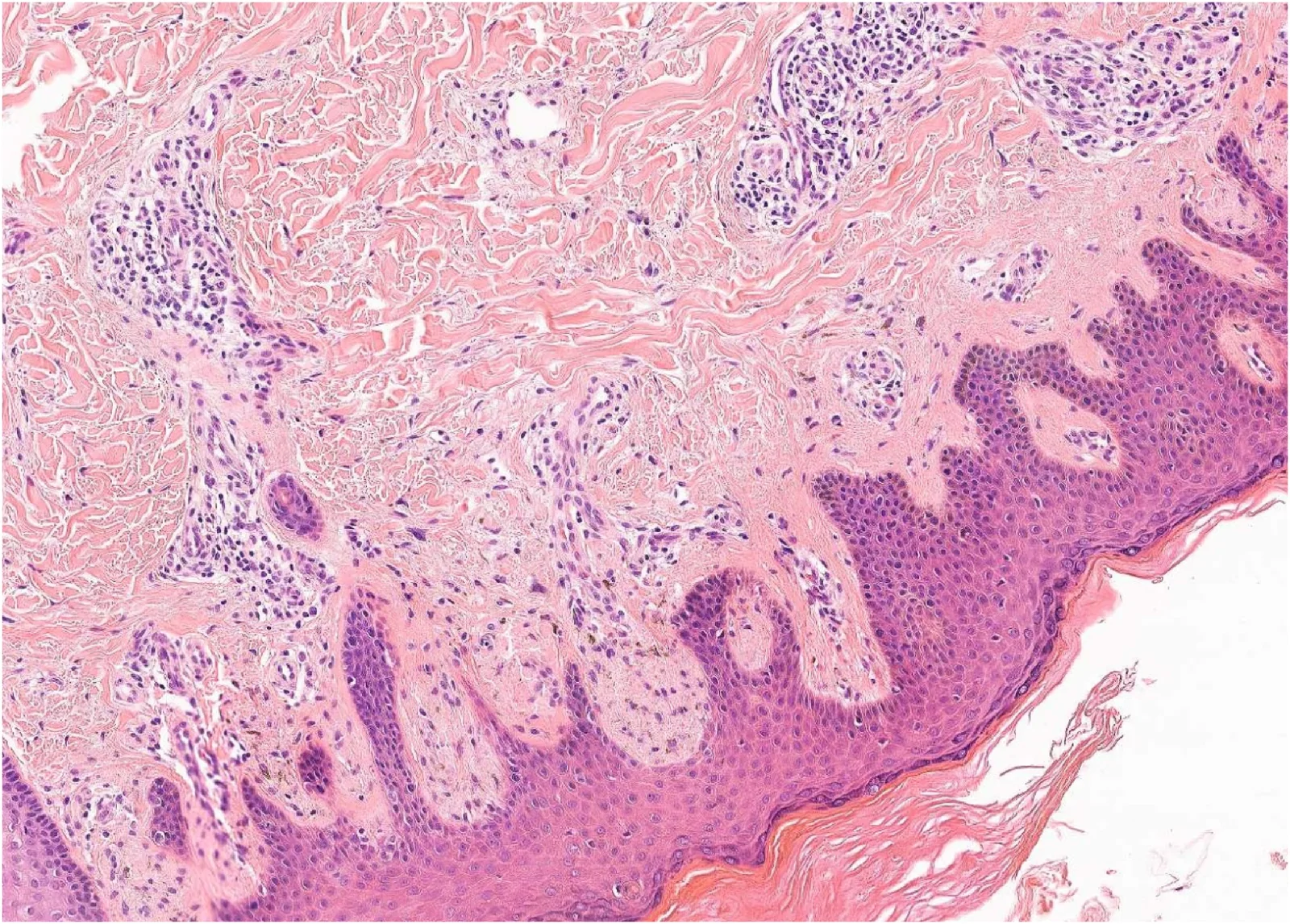
Histopathological findings of the lesion on the right lower leg (H&E ×100).

## Discussion

Lichen amyloidosis is a chronic, disfiguring, and pruritus-dominant dermatosis associated with significant impairment in quality of life. Its pathogenesis remains incompletely understood; however, chronic pruritus and repetitive scratching are widely regarded as central drivers of keratinocyte degeneration, amyloid formation, and lesion persistence. Notably, amyloid deposition in lichen amyloidosis is considered a secondary pathological consequence triggered by chronic scratch-induced keratinocyte injury, rather than the primary initiating cause of pruritus. In the present case, the definite initial trigger for chronic pruritus could not be fully identified. Nevertheless, accumulating evidence supports that IL-31-driven JAK1-associated signaling plays a critical role in initiating and amplifying pruritus, which sustains the self-perpetuating itch–scratch cycle and further promotes amyloid accumulation in the papillary dermis. This causal distinction is important for interpreting disease progression and the therapeutic effect of JAK1-targeted therapy. Emerging molecular evidence indicates that IL-31 is a pivotal cytokine in LA, being significantly overexpressed in skin lesions and capable of activating sensory neurons, via signaling through JAK1 and JAK2. This pathway directly induces intense itch and promotes local inflammation, making JAK1 a rational therapeutic target. Conventional therapies for LA are often ineffective in refractory cases. Antihistamines provide limited benefit because LA-associated pruritus is largely non-histaminergic. High-potency topical corticosteroids may temporarily reduce inflammation but rarely achieve sustained remission and carry long-term safety concerns (2). Biologic agents targeting the IL-4/IL-13 pathway (e.g., dupilumab) have been attempted in LA with mixed outcomes (3, 4). While some patients improve, others show minimal response, likely because dupilumab does not fully block IL-31 signaling (5). In contrast, JAK1 inhibitors such as upadacitinib directly suppress IL-31-mediated intracellular signaling, resulting in more rapid and robust control of pruritus (6–8). This mechanism explains the prompt symptom relief observed in our patient and aligns with previous reports of upadacitinib inducing remission in dupilumab-refractory LA. Our case demonstrates several key clinical observations: upadacitinib at 15 mg daily rapidly alleviates intractable pruritus and reverses cutaneous lesions in long-standing, treatment-refractory LA; clinical improvement can be observed as early as 1 month, supporting a fast onset of action; the drug is well tolerated with no observable short-term adverse events in this patient; and targeting JAK1-dependent cytokine signaling, particularly IL-31, interrupts the itch–scratch cycle and halts disease progression. Considering the well-documented risk of herpes zoster associated with JAK-inhibitor therapy, the patient received herpes zoster vaccination after initiating upadacitinib to reduce the potential risk of varicella-zoster-virus-related adverse events (9). In the present case, only total serum IgE was tested for allergological screening. Given the typical clinical manifestations and definitive histopathological diagnosis of lichen amyloidosis, and the absence of personal and family history of atopic diseases, specific IgE measurement, skin prick testing, and patch testing were not performed. Nevertheless, we acknowledge that a complete allergological work-up would further exclude atopic comorbidities that may contribute to chronic pruritus. Limitations of this report include its single-patient, uncontrolled design. Larger prospective cohort studies and randomized controlled trials are necessary to confirm the long-term efficacy, optimal dosage, treatment duration, and safety profile of upadacitinib in LA.

## Conclusion

This case confirms that oral upadacitinib at a dose of 15 mg once daily is a highly effective, rapidly acting, and well-tolerated therapeutic option for patients with refractory lichen amyloidosis. By potently inhibiting JAK1-mediated signaling—especially of the pruritogenic cytokine IL-31—upadacitinib breaks the pathological itch–scratch cycle, leading to significant improvement in both pruritus and skin lesions. Upadacitinib represents a valuable targeted therapy for patients who fail conventional treatments and may become a first-line systemic option for severe LA in the future. Further clinical research is warranted to validate these findings in larger populations.

## Data Availability

The original contributions presented in the study are included in the article/Supplementary Material; further inquiries can be directed to the corresponding author.
